# The Association of Retinoic Acid Receptor Beta2(RARβ2) Methylation Status and Prostate Cancer Risk: A Systematic Review and Meta-Analysis

**DOI:** 10.1371/journal.pone.0062950

**Published:** 2013-05-13

**Authors:** Tianyi Gao, Bangshun He, Yuqin Pan, Rui Li, Yeqiong Xu, Liping Chen, Zhenling Nie, Ling Gu, Shukui Wang

**Affiliations:** 1 Central Laboratory, Nanjing First Hospital, Nanjing Medical University, Nanjing, Jiangsu, China; 2 Department of Life Sciences, Nanjing Normal University, Nanjing, Jiangsu, China; Roswell Park Cancer Institute, United States of America

## Abstract

The retinoic acid receptor beta2(RARβ2) is a type of nuclear receptor that is activated by both all-trans retinoic acid and 9-cis retinoic acid, which has been shown to function as a tumor suppressor gene in different types of human tumors. Previous reports demonstrated that the frequency of RARβ2 methylation was significantly higher in prostate cancer patients compared with controls, but the relationship between RARβ2 promoter methylation and pathological stage or Gleason score of prostate cancer remained controversial. Therefore, a meta-analysis of published studies investigating the effects of RARβ2 methylation status in prostate cancer occurrence and association with both pathological stage and Gleason score in prostate cancer was performed in the study. A total of 12 eligible studies involving 777 cases and 404 controls were included in the pooled analyses. Under the random-effects model, the pooled OR of RARβ2 methylation in prostate cancer patients, compared to non-cancer controls, was 17.62 with 95%CI = 6.30–49.28. The pooled OR with the fixed-effects model of pathological stage in RASSF1A methylated patients, compared to unmethylated patients, was 0.67 (95%CI = 0.40–1.09) and the pooled OR of low-GS in RARβ2 methylated patients by the random-effect model, compared to high-GS RARβ2 methylated patients, was 0.54 (95%CI = 0.28–1.04). This study showed that RARβ2 might be a potential biomarker in prostate cancer prevention and diagnosis. The detection of RARβ2 methylation in urine or serum is a potential non-invasive diagnostic tool in prostate cancer. The present findings also require confirmation through adequately designed prospective studies.

## Introduction

Prostate cancer is the most commonly diagnosed noncutaneous neoplasia in the world. The disease predominantly affects men after the 6th decade of life and is associated with considerable morbidity and mortality [1]. Curative treatment entails radical prostatectomy or radiotherapy, and the best outcome is seen in patients with the earliest stage disease [2], [3]. Patients with locally advanced or systemic disease carry a poor long-term prognosis because of the notable lack of curative therapy [4].

Recently, methylation of CpG islands within the promoter and/or 5′ regions of genes is recognized as a common alteration in cancer-related genes often associated with partial or complete transcriptional disruption [5]. This epigenetic alteration provides an alternative pathway to gene silencing in addition to gene mutation or deletion, which is suggested to be a new molecular marker for early cancer detection [6]. The retinoic acid receptor (RAR) is a type of nuclear receptor that is activated by both all-trans retinoic acid and 9-cis retinoic acid [7]. There are three retinoic acid receptors (RAR), RARα, RARβ, and RARγ, which are differentially expressed during development and in adult life, and there is strong evidence that RARβ plays a central role in growth regulation of epithelial cells and in tumorigenesis[8]–[10]. The human RARβ gene generates multiple isoforms by use of promoters P1 and P2 and alternative splicing [11], [12]. P1 directs the transcription of isoform RARβ1, whereas P2 promotes the transcription of isoforms RARβ2 and RARβ4 [13].

The RARβ2 is mapped to chromosomal region 3p24 (−477/+392, GenBank accession numbers S82362 and M96016), which is expressed in most tissues and has been shown to function as a tumor suppressor gene in lung, breast, and gynecological neoplasia [14]–[17]. The RARβ2 promoter is characterized by a CpG (cytidine phosphate guanosine)-rich region, the CpG island [18], which is located in the 5′-untranslated region, along with several motifs that are potential binding sites for transcription factors such as AP-1, AP-2, and Sp1. Moreover, RARβ2 was shown recently to be frequently hypermethylated in several primary human neoplasms, including prostate cancer [19]–[21]. All of these findings suggested that it might play a pivotal role in the development of human cancer.

To date, RARβ2 methylation has been proved in a number of individual studies, which is detected not only in tissue samples but also in urine and serum samples. The prognostic value of RARβ2 methylation status in prostate cancer patient's diagnosis and the relationship between RARβ2 methylation and pathological stage of prostate cancer and Gleason score remains unclear. Therefore, a systematic review was performed of the literature with meta-analysis to obtain a more accurate evaluation of the role of RARβ2 methylation in prostate cancer management.

## Materials and Methods

### Publication selection

Studies were identified via an electronic search of PubMed and Google Scholar using the following key words: prostate cancer, PCa, retinoic acid receptor β2, RARβ2, RARbeta2, methylation. We also manually searched the references of these publications in order to retrieve additional studies. Only those published as full-text articles and in English were included as candidates. The search updated on 28, December,2012.

### Inclusion and exclusion criteria

Studies were selected for analysis if they met the following criteria: 1) they were original epidemiological studies on the correlation between RARβ2 promoter methylation and the prognosis of prostate cancer patients; 2) RARβ2 methylation status was examined using methylation-specific PCR (MSP) or quantitative MSP (QMSP); 3)the subjects in every study comprised patients and non-cancer controls; 3) studies should be with full text not only abstracts for relevant information extraction; 4) when the same patient population reported in several publications, only the most recent report or the most complete one was included in this analysis to avoid overlapping between cohorts; 5) the numbers of patients and controls in each study should be more than 5 respectively.

### Data collection

For each eligible study, we collected information regarding authors, year and source of publication, country of origin, inclusion criteria, exclusion criteria, pathological stage, Gleason score, RARβ2 methylation frequencies in non-cancer controls and patients of prostate cancer and the method for methylation detection. All included studies used non-cancer people as a control group, though some of them did not provide the definition of non-cancer. In studies defining non-cancer people, there are two definitions: (1) normal healthy person; (2) people with benign hyperplasia prostate. Since it is impossible to redefine non-cancer people on a unified standard, we combined non-cancer people in our meta-analysis according to their original group in each individual study. Of these studies, pathological stage ≤ T2 was defined as low-stage, and pathological stage ≥ T3 was defined as high-stage which were defined by clinical differentiation. Gleason score≥7 was defined as high-GS and Gleason score≤6 was defined as low-GS. The final eligible articles selected for further meta-analysis were evaluated independently by two reviewers. Minor discrepancies were resolved by the authors' discussion.

### Meta-analysis and statistical analysis

The foremost analysis examined the differences in the frequency of RARβ2 methylation between prostate cancer patients and non-cancer people by odds ratio (OR) with the corresponding 95% CI. Moreover, the strength of association between RARβ2 methylation and patients' pathological stage and tumor Gleason score were also assessed by OR with the corresponding 95% CI. To assess heterogeneity across the studies, the statistics analysis for heterogeneity was performed[22]. If the studies were shown to be homogeneous with P>0. 05 for the Q-statistics, the summary of OR was calculated by a fixed-effects model (the Mantel-Haenszel method) when between-study heterogeneity was absent [23]. Otherwise, a random-effects model (the DerSimonian and Laird method) was selected [24]. In addition, stratified analyses were also performed by material and method. Furthermore, a sensitivity analysis, by which a single study in the meta-analysis was deleted each time to determine the influence of the individual data set to the overall pooled OR, was performed to assess the stability of the results. The potential publication bias was examined visually in a funnel plot of log [OR] against its standard error (SE), and the degree of asymmetry was tested by Egger's test [25]. This meta-analysis was performed using the software STATA version 12. 0. All P-values were based on two-sided tests and a P-value of less than 0. 05 was considered statistically significant.

## Results

### Study characteristics

According to our inclusion criteria, a total of 12 eligible studies[21], [26]–[36] involving 777 cases and 404 controls were included in the pooled analyses. The characteristics of these studies are summarized in Table 1. Of these studies, two studies were conducted in Asia, three were in Europe, and the rest were in USA. The methylated RARβ2 levels were detected using either methylation specific PCR (MSP)[21], [32], [34]–[36] or quantitative methylation specific PCR (QMSP)[26]–[31], [33]. DNA methylation status of RARβ2 promoter was assessed in urine, serum or tumor tissues. Prostate cancer patients were confirmed pathologically in all the studies.

**Table 1:** Characteristics of studies included in this meta-analysis.

First author	Year	Location	material	Patient and control	Method	RARβ2 (M/U)^b^		P^a^(M/U)^b^		GS(M/U)^b^	
						case	control	Low-grade^c^	High-grade	Low-GS^d^	High-GS
Dumache R	2012	Romania	blood	91/94	QMSP	89/91	10/94	-	-	-	-
Bastian PJ	2007	Germany	tissues	78/30	MSP	56/78	1/38	32/47	24/31	30/48	26/30
Rouprêt M	2007	UK	urine	95/32	QMSP	59/95	1/32	-	-	28/55	31/40
Hanson JA	2006	USA	tissues	5/5	QMSP	4/5	0/5	-	-	3/18	8/20
Henrique R	2006	USA	tissues	30/30	QMSP	26/30	6/30	18/20	2/10	14/15	13/15
Hoque MO	2005	USA	urine	52/91	QMSP	18/52	8/91	7/24	11/28	11/24	7/28
Jerónimo C	2004	USA	tissue	118/30	QMSP	115/118	7/30	-	-	-	-
Karen Woodson	2004	USA	tissue	24/11	MSP	18/24	0/11	-	-	11/35	7/9
Jingmei Zhang	2004	USA	tissue	50/2	MSP	42/50	0/2	-	-	14/18	28/32
Yamanaka M	2003	Japan	tissue	109/36	MSP	85/109	0/36	29/39	56/70	33/47	52/62
Maruyama R	2002	USA	tissue	101/25	MSP	54/101	1/25	12/26	22/34	11/36	44/65
Nakayama T	2001	Japan	tissue	24/10	MSP	20/24	0/10	-	-	-	-

MSP, methylati on specific PCR; QMSP, quantitative methylation specific PCR. ^a^P pathologic stage; ^b^RARβ2 methylated/RARβ2 unmethylated; ^c^pathologic stage≤ T2 was defined as low-stage and pathologic stage≥ T3 was defined as high-stage; ^d^Gleason score≤6 was defined as low-GSand Gleason score≥7 was defined as high-GS.

### Meta- analysis

In general, the frequencies of RARβ2 methylation were tested in 12 reliable studies. The main results were summarized in Table 2. Under the random-effects model, the pooled OR of RARβ2 methylation in prostate cancer patients, compared to non-cancer controls, was 63.44 with 95%CI = 23.94–168.11. In the stratified analysis by material, significantly increased risks were found in tissues samples in detection RARβ2 methylation in prostate cancer(OR = 67.85, 95%CI = 33.75–136.42) and in non-tissues (OR = 46.76, 95%CI = 2.68–817.26). As stratified analysis by method, significantly increased risks were also found in MSP(OR 89. 15, 95% CI = 31.93–248.90) and QMSP (OR = 47.10, 95%CI = 10.59–209.53). In the evaluating the association between RARβ2 methylation and pathological stage in prostate cancer, study was carried out in five studies by fixed-effect model. The pooled OR of low-stage in RARβ2 methylated patients, compared to high-stage RARβ2 methylated patients was 0.67 (95%CI = 0.40–1.09, table 3). The relationship between RARβ2 methylation and tumor Gleason score was also compared by random-effect model. The pooled OR of low-GS in RARβ2 methylated patients, compared to high-GS RARβ2 methylated patients was 0.54(95%CI = 0.28–1.04, table 3).

**Table 2:** Stratified analyses of RARβ2 methylation and prostate cancer risk.

Variables	p^a^		OR		95% CI		Heterogeneity						
							X^2^		P		I^2^		
RARβ2													
total		12		63. 44		23.94–168.11		33.47		0. 001		67.1%	
total (trim-and-fill)		12		17.62		6.30–49.28		85.27		0.001		-	
material													
Non-tissue		3		46. 76		2.68–817.26		23. 18		0. 001		34.3%	
Tissue		9		67.85		33. 75–136.42		5.11		0. 746		0. 0%^b^	
method													
QMSP		6		47. 10		10.59–209.53		27.56		0. 001		81.9%	
MSP		6		89. 15		31.93–248.90		2.05		0. 842		0. 0%^b^	

a Number of comparisons.

b Between group heterogeneity not calculated; only valid with inverse variance method.

**Table 3:** Main results of eligible studies evaluating RARβ2 methylation and pathologic stage/Gleason score in prostate cancer.

Variables	p^a^	OR	95% CI	Heterogeneity		
				X^2^	P	I^2^
stage	5	0.67	0.40–1.08	1.75	0. 782	0.0%^b^
Gleason score	8	0. 54	0.28–1.04	17.91	0. 012	60. 9%

a Number of comparisons.

b Between group heterogeneity not calculated; only valid with inverse variance method.

### Sensitivity analyses

Sensitivity analysis revealed that 11 independent studies were the main source of heterogeneity[21], [26]–[29], [31]–[36]. Then the heterogeneity of RARβ2 methylation in prostate cancer patients, compared to non-cancer controls was decreased when Hoque MO's study was removed (P = 0.54). In addition, no other single study was found to impact the pooled OR as indicated by sensitivity analyses.

### Publication bias

**Figure 1:**
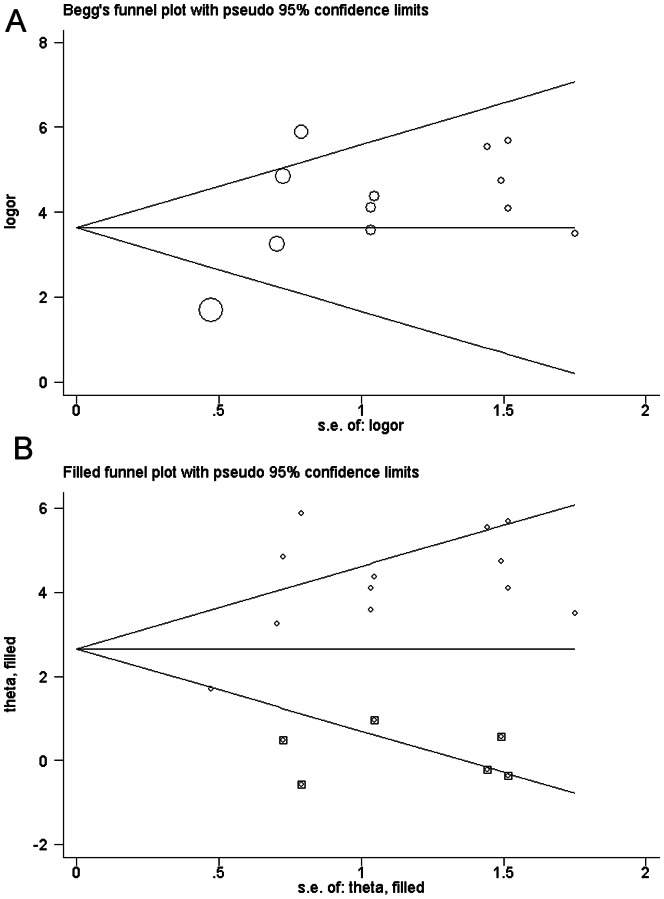
Begg's funnel plot with pseudo 95% confidence limits of publication bias test for RASSF1A methylation. Each point represented a separate study for the indicated association. Logor natural logarithm of OR, horizontal line mean effect size. A: Begg's funnel plot of publication bias test. B: Begg's funnel plot of publication bias test after trim-and-fill method.

**Figure 2:**
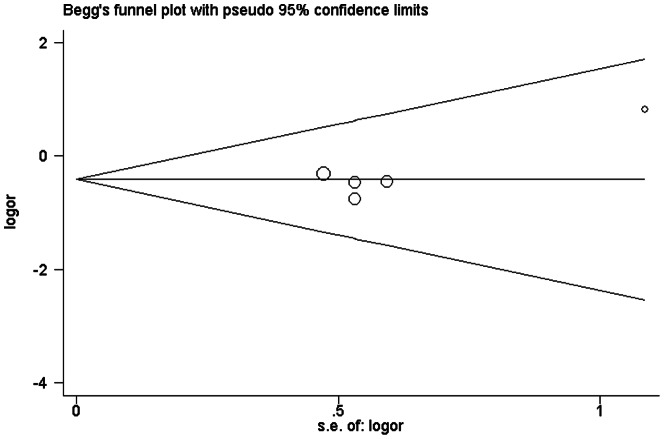
Begg's funnel plot with pseudo 95% confidence limits of publication bias test for association between RAR beta2 methylation and pathological state.

**Figure 3:**
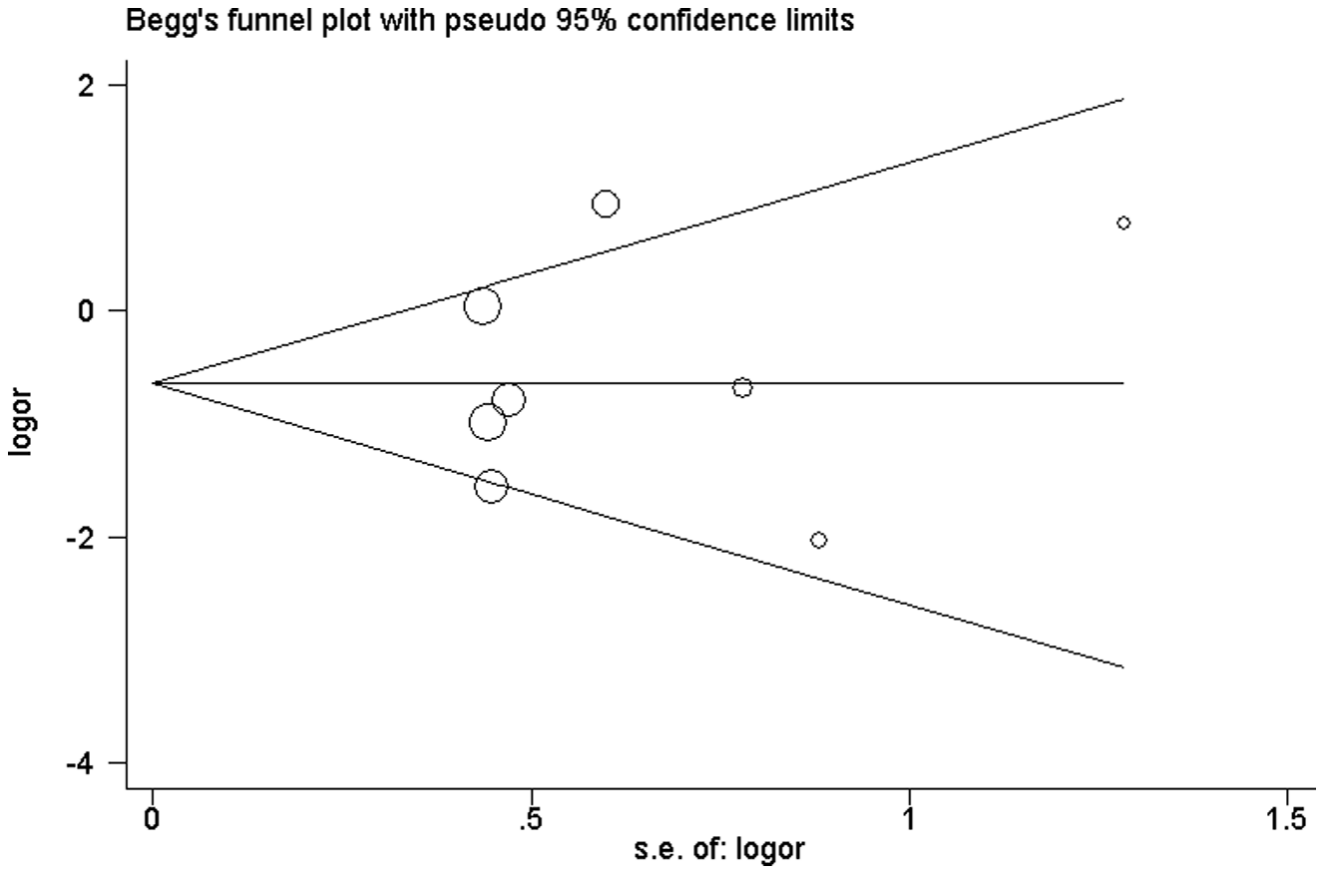
Begg's funnel plot with pseudo 95% confidence limits of publication bias test for association between RAR beta2 methylation and Gleason score.

As shown in Figure 1A, the shape of the funnel plots seemed asymmetrical in the methylation comparison between prostate cancer patients and non-cancer controls, suggesting the presence of publication bias. Then, the Egger's test provides statistical evidence of funnel plot asymmetry (t = 2. 38, P = 0. 038). To adjust this bias, a trim-and-fill method developed by Duval and Tweedie [38] was implemented (Figure 1B). The pooled OR of RARβ2 methylation in prostate cancer patients, compared to non-cancer controls, was 17.62 with 95%CI = 6.30–49.28 by trim-and-fill method (table 2). Meta-analysis with or without the trim-and-fill method did not draw different conclusions, indicating that our results were statistically robust. Funnel plot and Egger's test were performed to assess the publication bias in studies of association between RARβ2 methylation and pathological stage/Gleason score, The shape of the funnel plot did not indicate any evidence of obvious asymmetry (figure 2, 3) and the Egger's test suggested the absence of publication bias (P>0.05).

## Discussion

The results of our systematic review showed that RARβ2 methylation in prostate cancer was associated with tumor risk as either detected in urine, serum or tissue by MSP or QMSP. However, the RARβ2 methylation was not associated with increased risk for developing pathological stage or Gleason score of prostate cancer in comparison between RARβ2 methylated bladder cancer patients and unmethylated patients.

Hypermethylations of the RARβ2 gene having been reported in many studies declared that the frequency of RARβ methylation was found to be significantly higher in patients group compared with controls [21], [26]–[37]. Previous reports also demonstrated that genetic variations of RARβ affect prostate cancer susceptibility[39].To further confirm RARβ2 promoter methylation status in prostate cancer patient' s diagnosis, we carried out a meta-analysis of 12 studies involving 777 cases and 404 controls to derive a more precise estimation of the association. The analysis showed that RARβ2 methylation in prostate cancer patients, compared to non-cancer controls, was 17.62 times higher than that in non-cancer people after the trim-and-fill method which further confirmed RARβ2 methylation was a potential risk factor for prostate cancer as detected both in urine, serum and tumor tissues. MSP is a nonquantitative nonfluorometric PCR method to investigate promoter methylation. This method may fail to detect low concentrations of methylated alleles, unlike QMSP which can detect up to 1/1000 methylated alleles[40]. In this meta-analysis, both MSP and QMSP have the same effect in RASSF1A methylation detection.

However, Carmen Jerónimo's study suggested that RARβ2 methylation levels correlated with pathological tumor stage but not with Gleason score[32]. R. Dumache's study demonstrated that RARβ2 methylation was correlated not only pathological tumor stage but also Gleason score[26]. But in Woodson K's study, methylation of RARβ2 was proved to correlate with tumor grade but not pathological stage[41]. To resolve the conflicting results, we carried out a meta-analysis which indicated that the RARβ2 methylation status did not correlate with either the pathological stage or Gleason score of prostate cancer patients, suggested that inactivation of RARβ2 may be an early event in prostate carcinogenesis.

Early diagnosis of prostate cancer currently relied on trans-rectal ultrasound guided needle biopsy (10 to 12 cores) in men with increased total PSA (greater than 4.0 ng/ml) and/or abnormal DRE findings[42]. However, 65% to 70% of men with total PSA in the 4.0 to 10.0 ng/ml range had a negative prostate biopsy result[43]. In addition, more than 20% of men with PSA in the 2.0 to 4.0 ng/ml range were found to have cancer when evaluated by prostate biopsy[44]. More troublesome, there was no conclusive evidence that screening based on PSA decreases prostate cancer mortality[45]. Early detection of prostate cancer may be made more effective and efficient as RARβ2 may be an early biomarker in prostate carcinogenesis diagnosis.

Cancer is not a single cell disease. Aberrant cancer cells and their interactive microenvironment are needed for cancer to progress to androgen independence and distant metastasis[46]. Tumor heterogeneity in methylation patterns may be influenced by response to the microenvironment and local expression of genes, hormones, oxidative stress, or some other factor that can modulate methylation. Tsuyoshi Nakayama'study indicated that three CpG sites (numbers 20 to 22) near the βRARE region were consensus regions of methylation in PCa, which might be critical for the silencing of the gene by blocking access of liganded RAR/RXR heterodimers and other cis-acting transcription factors to their binding sequences[35].

RARb2 might be silenced not only by DNA methylation but also by histone deacetylation. Acetylation and deacetylation on lysine residues of histone amino-terminal tails had profound effects on gene transcription[47]. The RARβ promoter was under the control of a high-affinity retinoic acid response element itself. Thus, once silencing of RARβ had occurred, the lack of RAR-beta might reinforce the inactive silent state at its own promoter probably promoting methylation as a secondary repression mechanism[48]. Fuks F's study demonstrated that RARβ methylation-negative cells (LNCaP, PC3, and DU145) were hypo-acetylated at both H3 and H4. Combined TSA and all- trans retinoic acid treatment after 5-azacytidine treatment increases the accumulation of acetylated histones, leading to reactivation of the methylated RARβ promoter and subsequently the expression of RARβ[49].

In conclusion, our meta-analysis suggested that detection of RARβ2 methylation might be a potential biomarker diagnostic tool in prostate cancer. The detection of RARβ2 methylation in urine or serum is a potential non-invasive diagnostic tool in prostate cancer. It is necessary to conduct large sample size studies of the association between RARβ2 methylation and prostate cancer risk, eventually leading to our better understanding.
